# Accessing crop genetic diversity via pangenomics

**DOI:** 10.1007/s00122-026-05201-0

**Published:** 2026-03-13

**Authors:** Tessa R. MacNish, Venkataramana Kopalli, Silvia F. Zanini, Rod J. Snowdon, Agnieszka A. Golicz, David Edwards

**Affiliations:** 1 School of Biological Sciences, The University of Western Australia, Perth, WA 6009 Australia; 2 Centre for Applied Bioinformatics, The University of Western Australia, Perth, WA 6009 Australia; 3 Department of Agrobioinformatics, IFZ Research Centre for Biosystems, Land Use and Nutrition, Justus Liebig University, Giessen, 35392 Giessen, Germany; 4 Department of Plant Breeding, IFZ Research Centre for Biosystems, Land Use and Nutrition, Justus Liebig University, Giessen, 35392 Giessen, Germany

## Abstract

With the increasing accuracy and decreasing cost of sequencing technology, the extent of structural variation (SV) and its importance in crop species has become increasingly evident. SVs such as insertions, deletions, and inversions have been associated with genetic variation of agronomically important traits and the diversification of crop species. Pangenomes aim to capture the genetic diversity of a species, population or genus, by incorporating the genomes of multiple individuals. The additional genetic diversity represented by a pangenome compared to a single-genome reference can aid the association of variation with traits and support crop improvement. Genus-wide pangenomes representing related crop species as well as their wild relatives can be used to identify and introduce novel genetic variation associated with agronomically important traits into crops. Pangenomes can aid crop improvement through pangenome assisted breeding (PAB) and genome editing. PAB is an adaption of marker assisted breeding that associates pangenome-based markers, including single nucleotide polymorphisms (SNPs) and SVs, with a trait of interest. Genome editing can use CRISPR/cas9 or similar tools to introduce or change the expression of agronomically important SVs. Pan-epigenomics is an emerging field that can complement pangenomics studies by identifying epigenetic modifications such as DNA methylation, histone modifications, and chromatin accessibility, which play important roles in regulating gene expression and have been shown to contribute to intraspecific diversity and agronomically important traits. We highlight the advances of crop pangenomics and their use in crop breeding and improvement.

## Advances in DNA sequencing have revolutionised genomics

Our ability to read the genetic code of life has improved phenomenally over the last few decades, moving from manual radiolabelled Sanger-based DNA sequencing, through fluorescent labelled capillary based automated Sanger and then the introduction of massively parallel DNA sequencing (see Batley and Edwards 2016 for a comprehensive review). The first eucaryotic genomes were assembled using automated Sanger sequencing (Kaul et al. 2000; Lander et al. 2001), requiring multimillion-dollar investment over several years. As technologies improved it became feasible to assemble multiple reference genomes for a species and differentiate between sequence/assembly errors and true biological variation (Bayer et al. 2017). These early studies demonstrated the previously suspected high level of presence absence variation in plant genomes (Li et al. 2014). As the cost of DNA sequencing continued to plummet and the accuracy of long read DNA sequencing improved, it became possible to examine genome variation on a species wide scale revealing the full extent of variation (Yao et al. 2015), the mechanisms leading to sequence duplication and loss as well as the impact that this variation has in both natural and domesticated population (see Zanini et al. 2022 for a literature review). Pangenomes are multi-individual genomic resources and can capture this genomic variation for more comprehensive analysis than single reference genomes permit. As our understanding of genomics continues to grow, and with it, our ability to edit genomes, the potential for unprecedented scientific and societal advances becomes reality (see Tay Fernandez et al. 2022 for an in-depth review). Pangenomes will play an important part in this future as understanding the natural variation present in populations as well as the gene content of individuals to be edited are important for accurate and targeted genome modification.

## Structural variation has significant impacts on crop evolution and adaptation–early evidence to today

Early comparative analyses of bacterial artificial chromosome (BAC) contigs (Morgante et al. 2005) and array-based, comparative genomic hybridization data in maize (Springer et al. 2009), and of short-read whole-genome resequencing datasets from soybean (Lam et al. 2010; McHale et al. 2012) indicated that gene copy number and presence absence variation were widespread and frequently associated to adaptive traits. While transposon activity is known to play a notable role in local SV occurrence in maize, giving rise to widespread tandem gene duplications (Springer et al. 2009), SV patterns observed among ancestral gene homologues in maize and soybean also indicated a role of ancient polyploidisation events in SV formation, reflecting similar observations in Arabidopsis (Blanc and Wolfe 2004). This suggested a direct role of polyploidisation as a driver of gene diversification in crop evolution, although empirical evidence is difficult to establish in ancient polyploids.

Although large structural inversions can also have a significant impact on recombination and are hence important for breeding efforts, the vast majority of SV in most crop plants involve presence-absence variants (PAV) caused by local transposon activity, small-scale chromosome rearrangements or by more substantial chromosome exchanges, particularly in polyploid species. One of the first major examples demonstrating the significant evolutionary impact of gene PAV caused by large-scale homoeologous chromosome exchanges in the course of polyploid crop evolution was discovered when the first genome assembly of the recent allopolyploid crop, *Brassica napus* (oilseed rape, canola, swede/rutabaga) was completed (Chalhoub et al. 2014). Widespread homoeologous chromosome exchanges and gene conversions were subsequently discovered in the de novo*-*assembled and resequenced genomes (Chalhoub et al. 2014; Samans et al. 2017; Hurgobin et al. 2017). Since *B. napus* is a recent crop species thought to have arisen in the past few thousand years, after few isolated allopolyploidisation events (Lu et al. 2019) and subsequent outcrossing with its diploid ancestors, the overall level of genetic diversity in the species is expected to be limited. Limited diversity should inhibit evolutionary or breeding success. Paradoxically however, *B. napus* has become one of the most globally successful oilseed crop species and achieved broad ecotype diversification and ecogeographical adaptation to diverse climatic zones on all continents. A better understanding of the extent, mechanisms and consequences of de novo diversification through homoeologous genome interactions and rearrangements in *B. napus* gave rise to the hypothesis that widespread SV formation during and after polyploidisation is a driver of de novo crop diversity following the strong bottlenecks of polyploidisation and domestication, and that existing and ongoing SV formation enabled historical crop evolution and could be a key to future adaptation (Samans et al. 2017). Chawla et al. (2020) showed that SV alone can explain the diversification of *B. napus* into differentiated ecotypes and crop forms, while other studies linked specific intragenic deletions/insertions with disease resistance or flowering behaviour (Gabur et al. 2020; Vollrath et al. 2021a, 2021b). Later assemblies confirmed that the enlarged tuber of *B. napus* swede forms and the related tuberous morphotypes in the related allopolyploid *B. juncea* likely arose by direct human selection from the product of homoeologous chromosome exchanges that led to CNV among the genes responsible for this trait. Interestingly, SVs in *B. napus* have been shown to arise at a much higher rate than expected, with some genotypes having new variants arising in each generation (Orantes-Bonilla et al. 2022).

Beyond well-studied examples such as *B. napus*, the growing availability of high-quality genome and pangenome sequence assemblies for crop species is providing evidence to support a major role of genome-wide SV in crop evolution and diversification. An investigation of genome-wide gene PAV using exon capture across a panel of 434 wheat accessions revealed the presence of widespread chromosome introgressions, and gene loss or duplication caused by non-reciprocal homoeologous exchanges (Heuberger et al. 2024), reminiscent of the patterns reported in *B. napus,* and with related associations to ecogeographical diversification into groups with divergent phenological adaptation. Large chromosome introgressions were found to be frequent and to impact traits relevant for wheat adaptation and breeding, including important disease resistance traits. In many cases, introgressions originated from wild grass species. As in *B. napus*, evidence was also found that SVs affecting phenological genes have facilitated both global and local adaptation to ecogeographical regions (Jiao et al. 2025). Similarly extensive occurrence of gene presence absence variation as a consequence of homoeologous exchanges have also been reported in the hexaploid oat genome (Peng et al. 2022). Another example of this phenomenon was recently reported in natural populations of white clover (*Trifolium repens*), a globally important allopolyploid that has widespread natural populations and is also widely cultivated as a grassland crop. In an analysis of more than 2600 *T. repens* plants collected from six continents, (Battlay et al. 2024) revealed that large structural variants had imparted strong signatures of parallel adaptation to climatic zones.

Interestingly, new data from ancient polyploidisation examples indicate that polyploidisation is a strong evolutionary force under changing abiotic and biotic stress conditions due to its role in generating diversity (see van der Peer et al. 2021 for a comprehensive review). For example, whole-genome resequencing of 363 diverse accessions of sorghum, a major tropical cereal crop with a relatively small and simple diploid genome, showed that evolution into divergent crop use types and the emergence of variants for adaptation traits such as day-length are attributable to SV, some of which date back to ancient whole-genome duplication events (Zhang et al. 2024c). Overall, SVs were shown to have a disproportionate impact on trait variation, supporting a very early and ongoing association of SV with the emergence and selection of traits and thus potentially a major role in crop evolution. These results support work by Songsomboon et al. (2021), who observed cluster-specific deletions of genes related to biotic and abiotic stress responses and suggested a role of such deletions in local adaptation.

It is known that SV can readily occur through aberrant DNA repair after non-homologous end-joining, which requires only low- to medium-level sequence similarity at breakpoints (see the review by Saxena et al. 2014) and thus acts not only across interacting homoeologous chromosome segments but also in relation to tandem repeats. This may be an explanation for why abundant classes of resistance genes that are found in tandemly repeated clusters are frequently impacted by CNV, and why local tandem gene duplications are commonly observed in large crop genomes that have a high density of repetitive elements (Morgante et al. 2005; Jayakodi et al. 2023). Associations of SV impacting resistance gene clusters and corresponding disease traits are therefore not unexpected and have been reported in many important crops including soybean (McHale et al. 2012), sorghum (Zheng et al. 2011), rice (Fuentes et al. 2019) and tomato (see Andolfo et al. 2021 for a review).

The body of evidence supporting the impact of SV on crop evolution and adaptation continues to grow as pangenomic datasets and related trait observations are associated with these variants. The continuing discovery of trait-relevant SV and their implementation for quantitative genetic analyses and predictive breeding is likely to further increase our knowledge of their impact and utility throughout the coming decade.

## The development of pangenome methods

A pangenome captures the genetic diversity observed across a group of related individuals, aiming to be broadly representative of the wider population rather than exhaustive. This definition reflects that no pangenome can include every allele present in a species, but well-designed datasets provide a close approximation of population level diversity. The concept was first introduced in *Streptococcus agalactiae* (Tettelin et al. 2005), classifying genes as core (present in all individuals) and dispensable (absent in at least one).

Pangenomes capture complex genomic polymorphisms including structural variations (SVs), PAVs, and copy number variations (CNVs), key determinants of phenotypic variation across crop species. However, these resources often do not represent the full diversity within a species due to limitations in sampling, sequencing depth, or the difficulty of assembling repetitive sequences (see the reviews Sarashetti et al. 2024; Secomandi et al. 2025; Tørresen et al. 2019 for more details). Earlier studies focused mainly on gene level presence/absence matrices, whereas most current approaches use chromosome level assemblies that can represent large SVs and both genic and intergenic diversity. Chromosome scale pangenomes have now been constructed for several major crop species such as rice, maize, wheat, soybean, and rapeseed (Hirsch et al. 2014; Li et al. 2014; Montenegro et al. 2017; Song et al. 2020; Wang et al. 2018).

The following sections describe major methodological paradigms for pangenome construction, broadly categorized into linear and graph-based frameworks, as well as other approaches and are summarized in Table 1.

**Table 1:** Pangenome construction methods in both linear and graph-based pangenomes, highlighting their workflow, strengths, and limitations. Selected supporting studies illustrate their applications in plant research

	Method	Main steps	Strengths	Limitations	First application in plants
Linear Pangenome	Iterative Mapping & Assembly	Assemble genomes from multiple accessions → Map to reference → Call variants → Add novel sequences as unassigned contigs to the improved reference	Computationally efficient, good for small-scale studies	Limited detection of large SVs, reference-bias, Lack of contiguity /sequence context	(Golicz et al. 2016) *Brassica oleracea*
	De novo Assembly	Assemble genomes independently → Compare whole genomes → Call variants → Merge structural variations	Captures full genome diversity, unbiased by reference	Computationally intensive, requires high-quality long reads	(Y. Li et al. 2014) *Glycine soja* (Wild Soybean)
Graph Pangenome	Reference-based genetic variation graphs	Align assemblies to a reference → Call variants → Construct graph from the variants	Efficient for genotyping and variant discovery, captures known genetic variation	Limited to known variants, may miss novel variations	(Liu et al. 2020) *Glycine max* (Soybean)
					Tool: vg toolkit (Garrison et al. 2018)
	Reference-Based Pangenome	Align assemblies to a reference → Call variants → Construct variation graph from the variants	Preserves reference context, efficient for large datasets	Reference bias, may miss novel sequences	(Shang et al. 2022) *Oryza Sativa* (Rice)
					Tool: Minigraph (H. Li et al. 2020)
	Reference-Unbiased Pangenome	Perform all-vs-all alignment of assemblies → Call variants → Construct graph without reference	Captures all structural variations, suitable for diverse species	High computational cost, complex to interpret	No published plant study yet
	Reference-based Practical Haplotype Graph (PHG)	Define reference ranges → Upload haplotypes from assemblies → Call variants → Construct graph database	Efficient data storage, enables imputation for low-coverage sequences	Represents haplotypes instead of full sequences, visualization challenges	(Jensen et al. 2020) *Sorghum*

### Linear pangenome approaches

Linear pangenomes represent genetic diversity in a linear coordinate framework, extending or comparing assemblies to identify shared and variable regions. These methods are straightforward to implement and require relatively little computational power, but they cannot fully represent alternative haplotypes or large-scale structural rearrangements. Linear pangenomes can be generated either by an iterative mapping and assembly, or by comparison of independently assembled genomes; both differ from graph models, which encode multiple paths within a single coordinate space.

### Iterative mapping and assembly

The Iterative Mapping and Assembly strategy constructs a pangenome by repeatedly aligning reads from multiple individuals to a single reference, assembling unmapped reads, and integrating these sequences into the reference. This approach allows the reference to grow as more individuals are added, progressively revealing non reference genomic content. This strategy was implemented by Golicz et al. (2016) to generate the first *Brassica oleracea* pangenome, employing Bowtie2 (Langmead and Salzberg 2012) for read alignment, MaSuRCA (Zimin et al. 2013) for genome assembly and analysis, and SGSGeneLoss (Golicz et al. 2015) for gene PAV detection. Because outputs remain linear FASTA and VCF files anchored to reference coordinates, these workflows are directly compatible with conventional read mapping, variant calling, and gene annotation pipelines. The approach is computationally efficient and compatible with standard pipelines, but remains reference anchored and cannot capture large SVs or multiple haplotypes at a locus.

### De Novo assembly based pangenomes

Methods based on full de novo assemblies, independently assemble each genome and compare them to delineate core and dispensable regions, avoiding reference bias and enabling discovery of novel loci. Early implementations relied on short read assemblies that were fragmented and structurally incomplete. With the advent of long read and HiFi sequencing, as well as scaffolding methods such as Hi-C, optical mapping, and haplotype phasing, de novo assemblies became chromosome level and highly contiguous. This technological shift transformed de novo assembly-based pangenomes from fragmented collections of contigs into accurate, reference free genomic resources compatible with downstream graph-based analyses.

The first plant pangenome based on de novo assemblies, built for *Glycine soja* (Li et al. 2014), used short reads from seven individuals but was limited in structural resolution. This early example illustrated how short read data restricted detection of long insertions, tandem duplications, and other complex SVs. Building on this foundation, subsequent pangenome studies, for example the potato pangenome (Tang et al. 2022) leveraged HiFi sequencing and Hifiasm (Cheng et al. 2021) assemblies to construct high-quality genomes for comparative analysis. Each genome was annotated prior to pangenome construction using MAKER2 (Holt and Yandell 2011), after which gene models were compared across accessions to define core and variable loci. Subsequent analyses focused on disease-resistance genes, which were re-annotated and classified using nucleotide-binding repeat (NLR) gene annotations (Steuernagel et al. 2020).

### Graph based pangenome approaches

Graph based pangenomes represent genomic sequences and their variations in a non-linear structure. Nodes correspond to sequence segments, edges connect adjacent regions, and paths trace genome specific traversal (Zanini et al. 2022). These graphs encode sequence and structural variation in one unified framework, allowing direct mapping between linear and graph coordinates.

### Reference based graphs

Reference based graphs anchor variation to an existing reference genome and integrate new sequences via alignment and variant calling.

Reference based genetic variation graph approach applied in tomato pangenome project by Zhou et al. (2022) combined Minimap2 (Li 2018), a fast long-read aligner for mapping sequences to large genomes, for alignment, DeepVariant (Poplin et al. 2018), a neural network–based variant caller that infers SNPs and indels from read alignments, and multiple SV callers (Sniffles, SVIM, CuteSV) merged with SURVIVOR (Jeffares et al. 2017), a toolkit for merging and comparing structural-variant call sets. Graphs were built using the vg toolkit (Garrison et al. 2018), a software suite for constructing, manipulating, and mapping to variation graphs. The subsequent graphs can be used to genotype SVs in larger population sequenced with short reads. The use of high-quality long-read assemblies for the backbone better represents the complex genomic variation found in plants, while the use of short reads for genotyping reduces sequencing costs. However, as a reference-based method, genetic variation graphs are biased to previously detected variants and may overlook novel or complex SVs.

Minigraph (Li et al. 2020), extends this concept to hundreds of assemblies, aligning each to a reference to automatically detect insertions, deletions, and rearrangements. Graphs made using Minigraph can be scaled to large datasets such as in the reference-based pangenome graphs constructed from 251 rice assemblies (Shang et al. 2022). This method preserves reference coordinate systems and scales well with many genomes, but the drawback is that it remains biased toward the reference and may miss diverged or novel sequences not present in the anchor genome (Kopalli et al. 2025, accepted). Minigraph-Cactus (Hickey et al. 2024), which integrates the Minigraph and Cactus frameworks, first uses Minigraph to generate a guide tree that defines the order of genome alignment and then applies the Cactus multiple-genome aligner to build a hierarchical graph based on these alignments. This combination enables a more accurate incorporation of new sequences, retaining a larger proportion of non-reference sequence while preserving the reference coordinate system and improving representation of complex rearrangements. Although its application in plants is still emerging, a recent study in rice used Minigraph-Cactus to successfully resolve multiallelic tandem LTR-retrotransposon, demonstrating its utility for complex repetitive loci (Morales-Díaz et al. 2025).

### Reference unbiased graphs

Reference unbiased approaches treat all genomes equally and avoid anchoring variation to a single reference. The PanGenome Graph Builder (PGGB) (Garrison et al. 2024), constructs reference-free pangenomes through all-vs-all sequence alignments and graph induction, capturing a broad spectrum of structural variation without introducing reference bias. While successfully applied to human and bacterial datasets, its application to plant genomes is computationally intensive due to abundance of repetitive elements. To mitigate this, PGGB can operate at the chromosome level, reducing runtime and memory demands. This strategy enables detailed representation of complex rearrangements, although inter-chromosomal variation may be missed and the resulting graphs can be difficult to interpret because of their high connectivity. Benchmarking across plant datasets (Kopalli et al. 2025, accepted) showed that PGGB generates highly resolved graphs that capture extensive structural diversity, but at substantial computational cost. Benchmarking across plant datasets (Kopalli et al. 2025, accepted) showed that PGGB can capture extensive structural diversity, but at substantial computational cost. However, unlike Minigraph and Minigraph-Cactus, which maintain distinct paths for duplicated or repetitive segments, PGGB often merges copy-number variable regions such as segmental duplications and VNTRs into a single shared path traversed by multiple haplotypes, simplifying graph topology but reducing explicit repeat resolution (Liao et al. 2023).

### Comparative performance of pangenome graph construction methods

Studies in humans and cattle (Andreace et al. 2023; Leonard et al. 2023) have compared pangenome graph construction methods including Minigraph, Minigraph-Cactus, and PGGB. These studies found that Minigraph uses significantly less CPU, memory, and time than Minigraph-Cactus and PGGB, but does not capture small variants such as SNPs or short insertions and deletions and is affected by reference bias. Consequently, Minigraph-Cactus and PGGB retained up to five times more sequence from non-reference accessions (Leonard et al. 2023). PGGB constructed the most complex graphs, identifying the largest number of SNPs and haplotype paths, followed by Minigraph-Cactus and Minigraph (Andreace et al. 2023). PGGB also showed the most stable results across repeated runs, whereas Minigraph-Cactus graphs varied slightly and Minigraph graphs depended on the order in which accessions were added. While PGGB performs all-vs-all comparisons and removes reference bias, the entire graph must be rebuilt to incorporate new genomes, unlike Minigraph and Minigraph-Cactus, which can incrementally add assemblies.

Comparable benchmarking in plants has shown consistent trends. In this study by Kopalli et al. 2025, (accepted), systematic comparisons of Minigraph, Minigraph-Cactus, and PGGB using simulated and real-world assemblies in Sorghum bicolor demonstrated that pairwise callers such as SyRI (Goel et al. 2019) and SVIM-asm (Heller and Vingron 2020) achieved slightly higher precision in controlled simulations, but graph-based methods captured a broader range of structural variants, including complex and multiallelic events, in real-world data. Graph-based pangenomes built with Minigraph-Cactus and PGGB, also showed improved read-mapping accuracy compared with linear references. However, PGGB required extremely high computational resources; for example, building mapping indexes for a graph of seven Sorghum bicolor genomes needed over 2 TB of memory. The same study also extended benchmarking to soybean, barley and oilseed rape, where trends largely mirrored those observed in *Sorghum bicolor*.

Together, these findings demonstrate that the most appropriate pangenome graph construction method depends on the computational resources available and the intended downstream analysis.

Pangenomes have transformed population-scale genomics, improving variant detection, read alignment, and expression quantification (Yildiz et al. 2025). Current benchmarking in crops (Kopalli et al. 2025, accepted) indicates that while graph pangenome approaches outperform linear models in accuracy, challenges remain in scalability, computational efficiency, and graph interpretation. Emerging solutions such as rpvg (Sibbesen et al. 2023) show promise for transcript and population scale analyses.

### Other pangenome building approaches

The Practical Haplotype Graph (PHG) is a graph-based framework designed to represent genetic diversity using shared haplotypes instead of complete nucleotide-level assemblies. It captures variation as a network of consensus haplotype blocks that summarize sequence diversity across individuals, allowing efficient genotype imputation and genomic prediction. Developed for crop breeding (Jensen et al. 2020), the reference-based PHG method used in a sorghum pangenome study involved aligning sequence data to the sorghum BTx623 reference genome using BWA MEM (Li 2013). Variants were called with the HaplotypeCaller pipeline (Sentieon DNAseq, 2018), and the results used to construct consensus haplotypes. These haplotypes were clustered and condensed to form a compact set of consensus sequences. The pangenome graph is built by mapping these haplotypes using a hidden Markov model (HMM) and Viterbi algorithm (Rabiner 1989) and is stored in a relational database, allowing for efficient analysis and imputation of low-coverage genomic data. The PHG approach supports tasks such as genotyping and genomic prediction, helping to organize and analyze large genomic datasets (https://bitbucket.org/bucklerlab/practicalhaplotypegraph/wiki/Home).

PanTools (Sheikhizadeh et al. 2018) represents genomes using a generalized De Bruijn graph. It combines pangenome representation with functional and comparative genomics, enabling the storage and annotation of multiple genomes, orthology inference, and downstream analyses such as gene family clustering, functional annotation propagation, and synteny block comparison.

### Visualization tools for pangenome graphs

Visualization tools enable interpretation of complex pangenome graph structures. As pangenome graphs continue to increase in size and complexity, effective visualization is essential for exploring structural variants, inspecting graph topology, and validating genome alignments. These tools allow users to translate abstract graph data into interpretable visual formats, helping to identify genomic regions that differ between individuals and to assess connectivity within the graph.

ODGI (Optimized Dynamic Genome/Graph Implementation) (Guarracino et al. 2022) provides a comprehensive toolkit for graph manipulation and statistical analysis within GFA-based pangenomes. It supports editing, sorting, and dynamic updates of graphs, as well as interactive visualizations that facilitate the identification of genetic variation and haplotype structures. ODGI can also be used to edit graphs such as PGGB and Minigraph-Cactus by removing complex regions, ordering nodes, and merging segments; however, effective use of these features requires detailed knowledge of the tool’s functions, parameters, and the overall structure of the pangenome graph (Guarracino et al. 2022).

Panache, a web-based platform, provides an intuitive interface for visualizing pangenomes, enabling users to explore gene content, structural variants, and genome alignments (Durant et al. 2021). Panache requires pangenome graphs to be linearized prior to visualization, where each continuous string of sequence is represented as a linear block. By linearizing the graph pangenome, some SV information is lost; however, the linear pangenome gains a stable coordinate system, for reproducible navigation, and the graph pangenome can be more easily displayed and interpreted.

Other visualization tools have been developed to address specific challenges associated with pangenome graph interpretation. Bandage (Wick et al. 2015) provides an accessible graphical interface for viewing small to medium-sized GFA graphs, offering an overview of graph topology and connectivity, though it becomes less effective with large, complex plant genomes. More recently, PanGraphViewer (Yuan et al. 2023) introduced a dedicated interface for pangenome graphs, supporting GFA and GFAv2 formats, integration with annotations, and comparison of multiple individuals. Together, these visualization platforms provide complementary solutions that balance scalability, interactivity, and usability for exploring graph-based pangenomes.

### Future directions in pangenome construction

Taken together, the comparisons above show that the choice between linear, graph-based, and haplotype-based frameworks is driven by study goals, data quality, and available computational resources. Each approach offers complementary strengths: linear methods provide simplicity and compatibility with existing tools, graph-based methods capture complex structural variation and enable improved mapping, and haplotype-based frameworks such as PHG allow efficient imputation for breeding applications. However, all current strategies face challenges in scalability, standardization, and interoperability.

Progress in pangenomics therefore depends on improving the efficiency of assembly and graph construction, enabling incremental updates as new genomes become available, and developing visualization tools that are more user-friendly and capable of preserving structural and functional information. In parallel, consistent formats and annotation practices are needed so that gene models, SVs, and presence/absence information can be exchanged and compared across methods. Integrating genomic, transcriptomic, and epigenomic data within unified pangenome frameworks and leveraging haplotype-based approaches for downstream applications will help move plant pangenomics toward comprehensive, application-ready resources for biological discovery and crop improvement.

## Current state of pangenomics in crops

Graph-based pangenomes are considered the gold standard for pangenome construction because they can represent genome-wide haplotypes, store reference genome information alongside the variation between individuals, and represent multiple species, including wild relatives of domesticated crops (see Wang et al. 2023 for a review on the topic; Wang et al. 2024). Due to these benefits, an increasing number of graph-based pangenomes have been constructed for crop species including wheat, barley, tomato, and kiwifruit (Bayer et al. 2022a; Jayakodi et al. 2024; Li et al. 2023a; Wang et al. 2024). Bayer and colleagues (2022a) constructed a graph pangenome representing 16 bread wheat accessions and were able to identify the sequences that were conserved across all accessions, present only in some accessions, and unique to one accession. A graph pangenome representing 14 kiwifruit accessions found that 53% of gene families were variable between individuals (Wang et al. 2024). Within these SVs, the authors identified a variant in the promoter of *AcBCM,* associated with fruit color and increased chlorophyl accumulation. A graph pangenome in maize (Gui et al. 2022) found that 37.36% of SVs were in low linkage disequilibrium with SNPs indicating that these SVs represented genetic variation that could not be identified using SNPs alone. Additionally, these SVs had higher heritability scores and explained 14% more phenotypic variation than SNPs and were therefore more likely to be functionally important. A trait association analysis using the same maize graph pangenome (Gui et al. 2022) supported this conclusion, with 47.5% of gene PAVs significantly associated with an agronomically important trait. The additional heritability and the significant genomic regions associated with these SVs could not have been identified using a single genome reference or by the de novo pangenome constructed in the same study, demonstrating the importance of using graph pangenomes alongside single-genome and de novo pangenome references to identify all agronomically important genomic variation.

Genus-wide pangenomes aid crop improvement by identifying novel sources of genetic variation that can be introduced into crop species through hybridization and genome editing (see Khan et al. 2020 for a comprehensive review). Modern crop varieties have declined in genetic diversity (see Khoury et al. 2022 for a review on the topic), making these genus-wide pangenomes an important resource for crop improvement. In recent years, multi-species and genus-wide pangenomes have been constructed for major crops as well as for some minor crops (Table 2). A rice pangenome representing 16 domesticated and wild *Oryza* species identified an additional 63,881 gene families present in the wild relatives (Long et al. 2024). The pangenome consisted of 57% dispensable gene families, 33% species-specific gene families, and 10% core gene families. Disease resistance genes were found to be less diverse in the cultivated rice accessions than in their wild relatives, demonstrating that the wild rice accessions could be a novel source of disease resistance. A genus-wide pangenome of *Glycine* found that, after duplicated genes were removed, approximately 70% of the genes identified in the wild perennial relatives were not present in annual domesticated soybean (Zhuang et al. 2022). This *Glycine* pangenome was constructed with 26 accessions consisting of 8 domesticated and wild species and demonstrated novel genetic variation within wild relatives. Su and colleagues (2024) constructed an apple pangenome including cultivated and wild varieties. A total of 63% of gene family sets were dispensable, and the wild species gene annotations were enriched for traits including oxidative phosphorylation, pentose metabolic process, and response to salt. The cultivated apples had a greater number of disease resistance genes due to gene retention following repeat hybridization/introgression with multiple wild relatives. These studies demonstrate the ability of genus-wide pangenomes to identify novel genetic variation that can improve agronomically important traits and at the same time improve genetic diversity in crops through hybridization.

**Table 2:** Representative multi-species and genus-wide pangenome studies in crops and whether these pangenomes included accessions representing the wild relatives of the domesticated crop species

Genus	Species	Crop	Number of accessions	Pangenome type	Wild accessions	References
*Brassica*	*B. rapa, B. nigra, B. oleracea, B. napus, B. juncea,* and* B. carinata*		15	Iterative mapping and assembly	No	Z. He et al. 2021
*Brassica*	*B. napus, B.oleracea,* and *B. rapa*		41	Graph	No	MacNish et al. 2025
*Camellia*	*C. sinensis* and* C. assamica*	Tea	11	Gene-based	Yes	Tariq et al. 2024
*Cicer*	*C. arietinum, C.reticulatum, C. echinospermum, C. bijugum, C. judaicum, C. pinnatifidum, C. yamashitae, C. chorassanicum* and* C. cuneatum*	Chickpea	10	Graph	Yes	Khan et al. 2024
*Citrullus*	*C. naudinianus, C. colocynthis, C. rehmii, C. ecirrhosus, C. amarus, C. mucosospermus,* and* C. lanatus*	Watermelon	28	Graph	Yes	Yilin Zhang et al. 2024b
*Citrullus*	*C. lanatus, C. mucosospermus,C. amarus,* and* C. colocynthis*	Watermelon	547	Gene-based	Yes	Wu et al. 2023
*Cucumis*	*C. sativus* and* C. sativus*	Cucumber	12	Graph	Yes	H. Li et al. 2022
*Glycine*	*G. max* and* G. soja*	Soybean	1110	Iterative mapping and assembly	Yes	Bayer et al. 2022b
*Glycine*	*G. max, G. soja, G. falcata, G. stenophita, G. cyrtoloba, G. syndetika G. tomentella,* and* G. dolichocarpa*	Soybean	26	Gene-based	Yes	Zhuang et al. 2022
*Hordeum*	*H. bulbosum* and *H. vulgare*	Barley	33	Graph	Yes	Feng et al. 2025
*Hordeum*	*H. vulgare* and* H. spontaneum*	Barley	76	Graph	Yes	Jayakodi et al. 2024
*Lactuca*	*L. sativa, L. serriola, L. saligna, and L. virosa*	Lettuce	474	Iterative mapping and assembly	Yes	van Workum et al. 2024
*Malus*	*M. domestica, M. baccata, M. sieversii,* and* M. sylvestris*	Apple	12	Gene-based	Yes	Su et al. 2024
*Musa and Ensete*	*M. acuminata, E. ventricosum, M. itineransm, M. balbisiana, Musa x paradisiaca,* and* M. textilis*	Banana	15	Iterative mapping and assembly	No	Rijzaani et al. 2022
*Oryza*	16 species	Rice	17	Gene-based	Yes	Long et al. 2024
*Panicum*	*P. miliaceum*	Broomcorn millet	32	Graph	Yes	J. Chen et al. 2023a
*Pyrus*	*P. bretschneideri, P. sinkiangensis, P. communis, P. bretschneideri, P. betuleafolia, P. pyrifolia,*and *P. ussuriensis x communis*	Pear	7	de novo	Yes	Ding et al. 2024
*Solanum*	60 species	Potato	296	de novo	Yes	Bozan et al. 2023
*Solanum*	S.berthaultii, S. cardiophyllum, S. ehrenbergii, S. infundibuliforme, S. kurtzianum, S. microdontum, S. okadae, S. polyadenium, S. raphanifolium, and S. verrucosum, and S. violaceimarmoratum	Potato	50	Graph	Yes	Zhu et al. 2025
*Solanum*	*S. lycopersicum, S. lycopersicoides, S. habrochaites, S. pennellii, S. chilense, S. peruvianum, S. corneliomulleri, S. neorickii, S. chmielewskii, S. pimpinellifolium,* and* S. galapagense*	Tomato	13	Graph	Yes	N. Li et al. 2023a
*Sorghum*	*S. propinquum* and* S. bicolor*	Sorghum	16	Graph	Yes	Tao et al. 2021
*Spinacia*	*S. oleracea, S. turkestanica,* and* S. tetrandra*	Spinach	13	Gene-based	Yes	She et al. 2024
*Vitis*	29 species	Grapevine	72	Graph	Yes	L. Guo et al. 2025a
*Vitis*	*V. acerifolia, V. aestivalis, V. arizonica, V. berlandieri, V. girdiana, V. monticola, V. mustangensis, V. riparia,* and* V. rupestris*	Grapevine	18	Graph	Yes	Cochetel et al. 2023
*Zea*	*Z. mays, Z. nicaraguensis, Z. luxurians, Z. diploperennis,* and* Z. perennis*	Maize	721	de novo and Graph	No	Gui et al. 2022

The quality of alignments to graph and genus-wide pangenomes depends on the quality and relatedness of sequences, which can lead to short, low quality or highly divergent sequences being absent from the final pangenome. Minigraph (Li et al. 2020) requires strong colinear chains for alignment and can fail to align short or highly divergent sequences. A *Brassica* genus-wide pangenome constructed using minigraph (MacNish et al. 2025) found that *B. napus* and *B. oleracea* shared more sequence than the more closely related *B. napus* and *B. rapa* due to the lower quality of the *B. rapa* genomes used. In addition, the accession with the highest number of unique sequences was the reference genome ZS11, which could indicate reference bias. All other reference-based graph construction methods such as Minigraph-Cactus (Hickey et al. 2024) and PHG (Bradbury et al. 2022) risk failing to align the highly divergent sequences found in genus-wide pangenomes, due to reference bias. Genus-wide pangenomes constructed through reference-based iterative assembly (He et al. 2021; van Workum et al. 2024) share this limitation. Reference unbiased methods such as de novo pangenome construction may be better suited to identifying novel or highly divergent sequences. Gui and colleagues (2022) constructed a genus-wide maize graph pangenome which had a mapping rate of 99.4% and alignment identities of 91.1% for individuals not included in the pangenome, indicating that the graph pangenome represented the majority of genetic variation within the population. Therefore, genus-wide and graph pangenomes can represent the genetic repertoire of a population depending on the species and construction methods used. However, many pangenomes studies do not report the percent of sequence that is not represented by the pangenome, which makes it challenging to know which pangenome resources accurately represent the whole population and which methods are best for each species or genus.

## The application of pangenomes for crop improvement

**Fig. 1:**
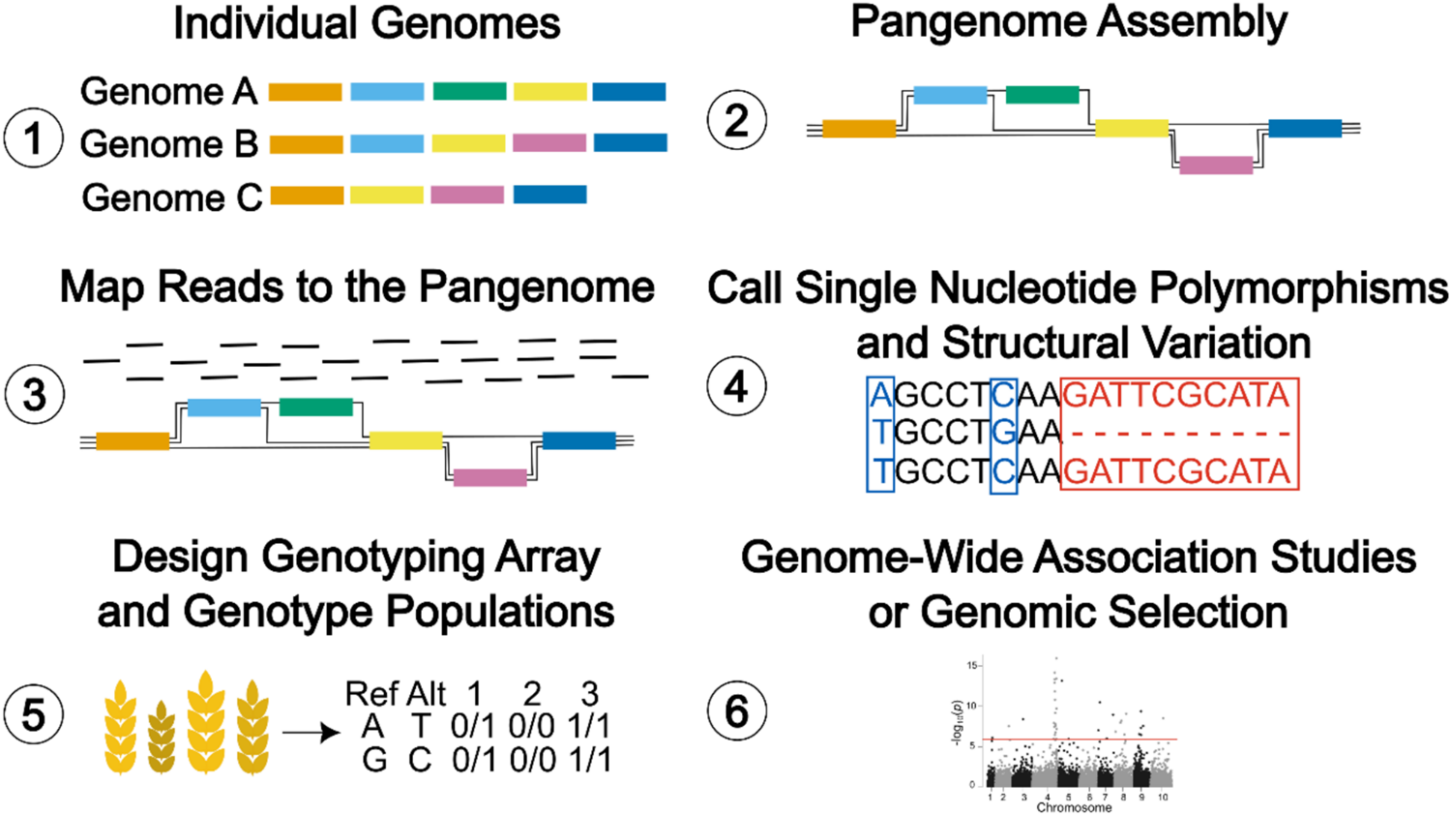
A workflow for the application of pangenomes for crop improvement. Genome assemblies of the crop of interest are collected and assembled into a pangenome. Pangenome-based genomic markers are identified by mapping reads to the pangenome and calling single nucleotide polymorphisms and structural variation. Once pangenome markers have been identified, they can be used to develop pangenome-based genotyping arrays. Markers identified by pangenome-based genotyping arrays can then be used in genome-wide association studies and genomic selection

The SVs identified in pangenomes contribute to gene expression and trait variation (Jeffares et al. 2017; Yuanyuan Zhang et al. 2024a) and have been significantly associated with agronomically important traits that can be used for crop improvement (Alonge et al. 2020). Pangenome assisted breeding (PAB) is an adaption of marker assisted breeding that associates pangenome-based markers, including SNPs and SVs, with a trait of interest. Pangenome-based markers can be identified by assembling a pangenome, mapping reads to the pangenome and calling SNPs and SVs (Chen et al. 2023a; Chen et al. 2023). Once pangenome markers have been identified, they can be used to develop pangenome-based genotyping arrays which efficiently, accurately and inexpensively genotype a population and provide markers for PAB methods such as pangenome-wide association studies (pan-GWAS) and genomic selection (GS) (Fig. 1). Current pangenome-based genotyping arrays include: BnaPan50T (Li et al. 2023b), a *Brassica napus* genotyping array with 54,765 markers for 588 accessions; rice pangenome genotyping array (RPGA) (Daware et al. 2023), including 271 rice accessions and 80 K markers; and *Triticum aestivum* next generation array (TaNG) (Burridge et al. 2024), a wheat genotyping array including 315 accessions and 43,372 markers.

Pangenome-based GWAS has been studied in several crops (Table 3). A study in sorghum (Tao et al. 2021) constructed a pangenome from 16 wild and domesticated accessions. The SNPs called from this were used in a GWAS analysis to identify 25 SNPs significantly associated with grain color. One of the most significant SNPs overlapped with a region of PAV that was associated with the known grain color gene *Yellow seed1*, identifying novel genetic variation associated with sorghum seed color that could not have been identified using single reference based GWAS alone. Chen and colleagues (2023) also found that pangenome-based GWAS could identify novel genetic variation for known causal genes. They used SNPs and SVs in a GWAS study of 43 agronomically important traits in broomcorn millet. The significantly associated SNPs and SVs overlapped. However, the significant SVs highlighted causal mutations associated with the traits of interest. For example, both significant SNPs and a significant SV were identified in *longmi008332*, a gene associated with inflorescence and seed color. The significant SV identified was an insertion that was found to be the causal mutation determining green or purple inflorescence. SV-based GWAS can be used together with SNP-based GWAS to examine genetic regions associated with agronomically important traits in greater detail to identify causal mutants and novel genetic variation. Pan-GWAS in tea identified both SNPs and SVs associated with leaf color, timing of bud flush, and flavor (Chen et al. 2023b). This study identified significant SVs overlapping with SNPs that were used to identify potential causal mutants, but they also identified significant SVs in novel genetic regions and found that only 9.1% of significant SVs overlapped with significant SNPs. These studies demonstrate the ability of pan-GWAS to not only identify potential causal mutations but to also identify novel sources of genetic variation that could be used to improve crops.

**Table 3:** Pangenome assisted breeding (PAB) studies in crops. The PAB studies include genome-wide association studies (GWAS) and genomic selection (GS) studies. These PAB studies used single nucleotide polymorphisms (SNPs) and/or structural variation (SV) markers to identify genomic regions and individuals associated with an agronomically important trait

Species	Crop	Pangenome type	Number of accessions	PAB method	Marker type	Trait studied	References
*Brassica napus*	Rapeseed	de novo	8	GWAS	SNPs and SVs	Silique length, seed weight, and flowering time	Song et al. 2020
*Brassica napus*	Rapeseed	Graph	57–100	eQTL	SNPs and SVs	Expression	Yildiz et al. 2025
*Camellia sinensis*	Tea	Graph	22	GWAS	SNPs and SVs	Leaf colour, timing of bud flush, and flavour-related chemistries	S. Chen et al. 2023b
*Cicer arietinum*	Chickpea	Iterative mapping and assembly	3202	GWAS	SNPs	16 traits	Varshney et al. 2021
*Cicer arietinum*	Chickpea	Iterative mapping and assembly	3202	GS	SNPs	9 traits	Varshney et al. 2021
*Citrullus*	Watermelon	Graph	28	GWAS	SNPs and SVs	Sugar content and flesh colour	Yilin Zhang et al. 2024b
*Cucumis sativus*	Cucumber	Graph	12	GWAS	SNPs and SVs	Female flower rate on a primary branch, fruit spine/wart density, and branch number	H. Li et al. 2022
*Glycine max*	Soybean	Iterative mapping and assembly	915	GWAS	SNPs	Flowering time	Mohamedikbal et al. 2024
*Lactuca*	Lettuce	Iterative mapping and assembly	474	GWAS	SVs	Disease resistance	van Workum et al. 2024
*Oryza sativa*	Rice	Graph	33	GWAS	SNPs and SVs	Leaf senescence	Qin et al. 2021
*Oryza sativa*	Rice	Iterative mapping and assembly	3010	GWAS	SNPs	Grain size/weight traits	Daware et al. 2023
*Panicum miliaceum*	Broomcorn millet	Graph	32	GWAS	SNPs and SVs	43 traits	J. Chen et al. 2023a
*Pennisetum glaucum*	Pearl millet	Graph	11	GS	SNPs and SVs	120 traits	Yan et al. 2024
*Setaria italica*	Foxtail millet	Graph	110	GWAS	SNPs and SVs	68 traits	Q. He et al. 2023
*Setaria italica*	Foxtail millet	Graph	110	GS	SNPs and SVs	68 traits	Q. He et al. 2023
*Solanum*	Potato	Graph	50	GWAS	SVs	Metabolite content	Zhu et al. 2025
*Solanum lycopersicum*	Tomato	Graph	31	GWAS	SNPs and SVs	Fruit soluble solids content	Zhou et al. 2022
*Solanum*	Tomato	Graph	13	GWAS	SNPs	Flavour-related metabolite traits	N. Li et al. 2023a
*Sorghum*	Sorghum	Graph	16	GWAS	SNPs	Grain colour	Tao et al. 2021
*Sorghum bicolor*	Sorghum	Graph	24	GS	SNPs	Height, brix, juice weight, leaf weight, ear-liness, stem weight, and grain yield	Jensen et al. 2020
*Spinacia*	Spinach	Gene-based	13	GWAS	SNPs and SVs	sex	She et al. 2024
*Spinacia*	Spinach	Gene-based	13	GS	SNPs and SVs	17 traits	She et al. 2024
*Triticum aestivum*	Wheat	k-mer-based	139	GWAS	SVs	Seed-storage proteins	Z. Zhang et al. 2024d
*Triticum aestivum*	Wheat	k-mer-based	139	GS	SVs	Seed-storage proteins	Z. Zhang et al. 2024d
*Vitis vinifera*	Grapevine	Graph	29	GWAS	SNPs and SVs	29 traits	Z. Liu et al. 2024b
*Vitis*	Grapevine	Graph	18	GWAS	SNPs and SVs	Chloride exclusion	Cochetel et al. 2023
*Zea*	Maize	Graph	507	GWAS	SNPs and SVs	Agronomic traits, metabolites, and protein contents	Gui et al. 2022

Recently, Yildiz et al. (2025) reported the first application of graphical pangenomics approaches for expression quantitative trait locus (eQTL) analysis in crops. A pangenome graph was built and used as reference for both genotyping and transcript expression quantification of 100 winter *Brassica napus* lines. Pangenome graph-based transcript quantification proved more accurate than linear-based, with transcripts with medium/low correlation between approaches being significantly overrepresented in SNPs and SVs. Noticeably, approximately a third of the transcripts associated with eQTL-SVs had no significant association with SNPs, highlighting the importance of including SVs in association studies and the added value of pangenome graph-based eQTL analyses. Altogether, this work resulted in the identification of hundreds of gene proximal eQTL-SVs, with several variants affecting stress response- and morphogenesis- related transcripts levels, pointing to a potential role of SV-driven expression variation in phenotypic diversity and trait fine-tuning in rapeseed.

The use of pangenome-based markers in GS has been found to increase trait prediction accuracy in several crops (Table 3). A GS study in pearl millet used both SNPs and SVs to identify breeding candidates for 120 agronomically important phenotypes (Yan et al. 2024). The SNP-based GS identified 9 breeding candidates while the SV-based GS identified 4. The SV-based GS captured genetic diversity not identified by SNPs and had higher prediction accuracy, demonstrating the benefit of using both SNPs and SVs in GS. Studies in tomatoes and grapevine found a 24% and 22.78% increase in estimated heritability when using a pangenome compared to a single reference genome, respectively (Liu et al. 2024b; Zhou et al. 2022). This increase in heritability due to the inclusion of SVs was credited for improving the power of GWAS to identify significantly associated markers and for the increase of trait prediction accuracy. A similar study in spinach supports the conclusion that capturing missing heritability with SVs improves trait prediction accuracy (She et al. 2024) and that PAB can improve the accuracy and efficiency of breeding programs compared to single genome reference based assisted breeding.

Genome editing is an alternative to conventional breeding methods for introducing SVs into crops. Sun et al. (2024) used CRISPR/Cas9 to successfully insert, delete, and translocate SVs of up to 30 Mb in rice. Similarly, Y. Lu et al. (2021) improved herbicide resistance in rice without affecting other agronomic traits, by generating a 911 kb inversion on chromosome 1 and a 338 kb duplication on chromosome 2. These studies highlight how genome editing can use SVs for crop improvement.

## Pan-omics

Pan-epigenomics is an approach seeking to characterize epigenetic variation within a species, complementing pangenomics and pan-transcriptomics with the study of epigenetic modifications such as DNA methylation, histone modifications, and chromatin accessibility. These modifications play an important role in regulating gene expression, transposable element activity, and developmental processes, and have been shown to contribute to intraspecific diversity and several crop traits, including stress tolerance, flowering behavior and yield (Crisp et al. 2016; Eichten et al. 2013; Guo et al. 2023; Lieberman-Lazarovich et al. 2022; Vafadarshamasbi et al. 2022; Xu et al. 2020, 2019)(See Crisp et al. 2016; Lieberman-Lazarovich et al. 2022; Vafadarshamasbi et al. 2022 for reviews on the topic). Complementarily, structural variations can disrupt gene structures, alter both near and distal regulatory sequences, or reposition transposable elements, thereby affecting local and global epigenetic landscapes (see Noshay and Springer 2021; Zhang et al. 2018 for literature reviews). This non-coding variation can be the basis for elucidating molecular mechanisms and regulatory networks in plants and also provide novel sources for fine-tuning crop genetic improvement (see Crisp et al. 2022; Ganguly et al. 2022; King et al. 2010; Lieberman-Lazarovich et al. 2022; Zhi and Chang 2021 for reviews discussing the topic).

While plant pan-epigenomic studies are only at their infancy due to the previously prohibitive costs of assessing genome-wide epigenetic features for multiple individuals, recent advancements such as single-molecule real-time sequencing and nanopore-based methylation detection have enabled high-resolution epigenomic profiling. Notably, Noshay and colleagues (Noshay et al. 2021) investigated the variability of both chromatin accessibility and DNA methylation across four maize inbred lines with high-quality reference genomes available. This study revealed that, while only 5% of unmethylated regions (UMRs) were polymorphic between genotypes, more than 70% of the conserved UMRs exhibited incomplete overlaps, even in the absence of underlying sequence variation. Similarly, the majority of accessible chromatin regions (ACRs) were conserved between genotypes, but almost 29% of accessible UMRs in one accession were found unmethylated and/or inaccessible in the other genotypes. This previously underexplored epigenetic variability could be an important contributor to intraspecific differences with functional consequences.

Recently, Zhao et al. (2024) generated population-wide DNA methylation data and integrated this with both genetic polymorphism and transcriptomic data for 207 cotton accessions. Association analyses identified 5.4 million cis-methylation quantitative trait loci (cis-meQTLs), and 5000 cis-expression quantitative trait methylations (cis-eQTMs), with approximately 36% of cis-eQTM genes not found associated with genic variations. Notably, one cis-eQTM gene that was not identified by GWAS alone was validated via gene editing, proving its role in fiber development. These results establish the potential of DNA methylation variation for identifying novel opportunities for crop improvement.

Pan-transcriptomics is another emerging field, with the development of sequencing technology making multi-tissue and multi-environmental sampling more accessible. A pioneering work from 2014 investigated a maize pan-transcriptome in a panel of 503 inbred lines at the seedling stage, enabling the identification of transcript abundance and transcript PAV associated with the transition between juvenile to adult growth and with flowering time (Hirsch et al. 2014). More recently, a pan-transcriptome of 20 barley individuals was constructed using short- and long-read RNA-sequencing along with Iso-seq data (Guo et al. 2025b). The barley pan-transcriptome improved mapping rate and found more transcripts per gene compared to a single-genome reference. Analysis of gibberellic acid (GA) metabolism, using the pan-transcriptome, found differential expression of GA genes including the overexpression of *GA2ox3*, which was predicted to improve yield under environmental stress (Guo et al. 2025b). A rice pan-transcriptome found an increased number of multiple splicing isoforms compared to a single-genome reference (Zhong et al. 2024). Zhong and colleagues (2024) constructed the pan-transcriptome from a variety of cultivars and cold stress conditions and identified cultivar- and cold stress-specific transcripts and alternative splicing cites. It was found that differentially expressed genes and alternative splicing-regulated gene expression were associated with cold stress tolerance, which varied between cultivars (Zhong et al. 2024). These results could inform breeders of which cultivars are better adapted to cold stress.

Alternative splicing (AS) events are known but largely unexplored sources of intraspecific diversity, with an estimated 20% of genes having one or more alternative transcript isoforms in plants (see Barbazuk et al. 2008 for a review). To our knowledge, there are no pan-multiomics studies that focus on alternative splicing events in plants, despite studies finding DNA methylation and the epigenome play an important role in affecting alternative splicing events for agronomically important traits under environmental stress conditions (Qian et al. 2019; Wang et al. 2021). Nevertheless, novel tools are being developed to investigate AS events using pangenomes, namely the *pantas* toolkit that combines a genomic variation graph with short-read RNA sequencing data to annotate the graph pangenome with alternative splice event information (Ciccolella et al. 2024). Pantas outperformed alternative splicing site identification tools based on a single-genome references (Ciccolella et al. 2024). This tool can combine genomic and transcriptomic data to identify alternative splice sites that are associated with agronomically important traits. Future studies should develop pangenome methods that can incorporate transcriptomic and epigenomic data alongside genomic data, such as pantas. Additionally, further research should be done on the effect of the transcriptome and epigenome on alternative splicing events associated with agronomically important traits.

Given these promising case studies pan-transcriptome and pan-epigenome atlases, profiling gene expression and epigenetic marks across a wide range of genetic backgrounds with high-quality reference genomes, could prove instrumental (see Huang et al. 2022; Jayakodi et al. 2021 for reviews on the topic). These atlases will be instrumental in deciphering the interplay between genetic variation, epigenetic modifications, and environmental factors in shaping phenotypic diversity.

Developing user-friendly pangenome browsers with integrated transcriptomics and epigenetic data will be useful for making this complex information accessible to researchers and breeders. Existing platforms such as Panache (Durant et al. 2021) and PPanG (Liu et al. 2024a) visualize pangenomes alongside individual sequences and tracks with gene annotation, coordinates, and alignments. Visualization tools such as these could be adapted to display additional tracks for UMRs and ACRs. Users could then select different environmental conditions and visualize methylation changes for each individual. Additionally, for epigenomic markers found to be associated with important traits, functional annotation could be displayed. To our knowledge, no pangenome browsers have integrated visualization for epigenetic information; however, these visualization tools would support comparative epigenomics, allowing users to track epigenetic changes across different accessions and conditions. Such resources will enhance the application of pan-epigenomics in precision breeding, supporting the selection of genotypes with desirable epigenetic traits for improved yield, stress tolerance, and adaptability.

Overall, pan-epigenomics and pan-transcriptomics represent promising frontiers in plant genomics, expanding our understanding on how genetic epigenetic, and transcriptomic variation collectively shape phenotypes. By expanding our understanding of epigenetic and transcriptomic diversity within and between species, these fields have the potential to revolutionize crop improvement, ecological adaptation studies, and functional genomics research.

With the growth of multi-omic studies, pangenomes could be combined with other omics such as metabolomics and proteomics to avoid reference bias also in these investigations. A PAV-based metabolomics-GWAS in potatoes identified 9321 SVs associated with 1258 distinct metabolites (Zhu et al. 2025). These metabolites affected agronomically important traits such as starch and flavonoid content and were not identified by SNP-based GWAS. A combined genomic and pan-metabolic study in cassava found that genetic and leaf metabolite subgroups correlated with eco-geological regions, while root metabolite clades did not (Perez-Fons et al. 2023). In addition, genetic subgroups correlated with agronomically important traits such as dry matter content, cooking time, and cyanide content but not to whitefly tolerance, while leaf metabolites did correlate with whitefly tolerance. These studies highlight how genomics and metabolomics can identify different sources of variation. Combining pangenomics with other omics generates a more complete picture of crop systems and can be used to identify the causal genetic/epigenetic variants associated with agronomically important traits. Currently, there are pangenome-based multi-omics databases for key crops such as *Brassica napus* (Cui et al. 2023), cereals such as oat and barley (Yao et al. 2022), and potatoes (Zhu et al. 2025). *Asteraceae* multi-omics information resource (AMIR) is a database containing 74 *Asteraceae* species and corresponding genome, pangenome, functional annotation, variants, ortholog, and tissue specific transcriptome data (Liu et al. 2025). AMIR additionally includes bioinformatic tools such as BLAST and Gene Ontology to streamline bioinformatic analyses within *Asteraceae* crops. Liu and colleagues (2025) demonstrated the use of AMIR by searching their pangenome for a known agronomically important gene, confirming the gene was a core gene, identifying homologous genes, and constructing a phylogenetic tree of homologs. More databases such as these could be collated and made public to foster global collaboration and innovation.

## Conclusion

The decreasing cost and increasing quality of sequencing data is driving the development of more pangenomes and expanding the scope of comparative pangenomics. Previously, genes were considered lost if they were missing from a single reference, however now we can measure the frequency of gene presence in a population. This frequency measure can be compared within and between species to help us understand selection at both the individual and population scale. Several minor crops and many wild plant species have not benefitted from pangenome analysis. Minor crops and wild relatives are an excellent source of genetic variation that can be used to improve the major crop species and also support the domestication of new species to expand the diversity of food crops. Crop pangenomics is a rapidly developing research area that can improve our understanding of the genetic diversity underlying agronomic traits; this knowledge can be used to improve crops through pangenomics assisted breeding and genome editing.
